# N-acylation of ethanolamine using lipase: a chemoselective catalyst

**DOI:** 10.3762/bjoc.5.10

**Published:** 2009-03-25

**Authors:** Mazaahir Kidwai, Roona Poddar, Poonam Mothsra

**Affiliations:** 1 Green Chemistry Research Laboratory, Department of Chemistry, University of Delhi, Delhi-110007, India

**Keywords:** chemoselective, lipase, microwave irradiation, zein number

## Abstract

The N-acylation of ethanolamine (**2**) with various fatty acids **1a**–**d** and esters of fatty acids **1e**–**h** using *Candida antarctica* B lipase (Novozym^®^ 435) are described and optimum conditions for selective N-acylation rather than O-acylation are also discussed. Microwave assisted solution phase, solid supported and conventional methods were investigated and results were compared. There is a synergy between the enzyme catalysis and microwave irradiation.

## Introduction

Microwave assisted enzyme catalysis is a popular developing methodology in the field of green chemistry [1]. Enzymes have gained pivotal importance in the production of fine chemicals, pharmaceuticals and numerous other manufactured goods [2–3]. These reactions represent new low cost processes which allow the use of non-toxic starting materials. Lipases are the most widely used enzymes because they are cheap, easily available, cofactor free and have broad substrate specificity [4]. Lipases (triacyl glycerol hydrolases EC 3.1.1.3) catalyze hydrolysis, esterification, transesterification, thioesterification, amidation, epoxidation etc. [5–10]. The use of immobilized lipases is on the rise, as these work well with non-aqueous media [11]. Apart from the convenient handling, these reactions also have the two-fold benefit of easy separation of the enzyme from the product and reusability of the enzyme [12–13].

Since the last decade, researchers have attempted to use microwave irradiation (MWI) to improve enzymatic reactions, an area which has been reviewed recently [14]. Microwave irradiation has advantages over conventional heating (CH), in the control of reaction rate, yield, stereoselectivity or chemoselectivity [15]. Microwave assisted heating under controlled conditions is an invaluable technology for organic synthesis because it often dramatically reduces reaction times, typically from days or hours to minutes or even seconds. Many reaction parameters, such as reaction temperature and time, variations in solvents, additives and catalysts, or the molar ratios of the substrates, can be evaluated in a few minutes to optimize the desired reaction. It is worth noting that microwave not only affects the selectivity but also the purity of the product [16].

Biosurfactants, an important class of compounds containing amide bonds, are potential substitutes of emulsifiers, which may be toxic [17–18]. Surfactants enter the environment in significant amounts through wastewater treatment plants or by direct release into watercourses. Their possible chronic effects on sensitive species and their potential environmental impact must be minimized, depending on the biodegradability and toxicity of the surfactants. Potential mineralizing under aerobic and anoxic conditions is important for hazard assessment of a surfactant because degradation often reduces the toxicity of these chemicals [19].

The hydroxy-substituted aliphatic amides **3a**–**d** act as surfactants, synthesized from fatty acids or esters **1a**–**h** and ethanolamine (**2**) and they are highly acceptable to the environment as they are characterized by their skin tolerance, good biologic degradability and low toxicity [20]. They also reduce the zein number, which is equivalent to a better skin tolerance.

Keeping in view the above benefits of hydroxy-substituted aliphatic amides **3a**–**d**, it was thought worthwhile to perform their synthesis using lipase (CAL B Novozym^®^ 435) with fatty acids or esters **1a**–**h** and ethanolamine (**2**) under simultaneous microwave irradiation.

## Results and Discussion

**Scheme 1:**
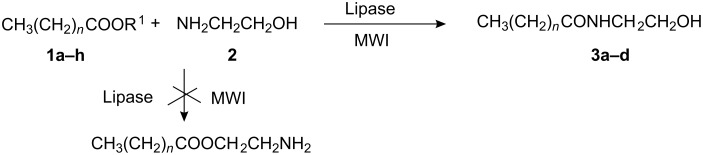
**1a** *n* = 8 R^1^ = H; **1b** *n* = 10 R^1^ = H; **1c** *n* = 12 R^1^ = H; **1d** *n* = 14 R^1^ = H; **1e** *n* = 8 R^1^ = C_2_H_5_; **1f** *n* = 10 R^1^ = C_2_H_5_; **1g** *n* = 12 R^1^ = C_2_H_5_; **1h** *n* = 14 R^1^ = C_2_H_5_; **3a** *n* = 8; **3b** *n* = 10; **3c** *n* = 12; **3d** *n* = 14.

In the reaction of fatty acids **1a**–**d** or esters of fatty acids **1e**–**h** with ethanolamine (**2**), two types of acylated products are possible. Out of two possible acylations, O-acylation and N-acylations, the latter is more favoured in aminoalkanol NH_2_(CH_2_)*_n_*OH because the amino residue serves as a better nucleophile than the hydroxy group. The selectivity of the aminoalkanol NH_2_(CH_2_)*_n_*OH towards N-acylation rather than O-acylation is attributed to following reasons (i) the high reactivity of primary amine, (ii) the fact that use of excess fatty acid or ester **1a**–**h** is not required to convert virtually all the amine to its ion-pair form and (iii) in aminoalkanols NH_2_(CH_2_)*_n_*OH, where *n* < 3, there is migration of acyl groups from O→N spontaneously when excess of fatty acid or ester **1a**–**h** is used, while in cases where *n* ≥ 3 the reverse occurs [21–22] (Scheme 1).

The identification between two possible products was done on the basis of ^1^H NMR. For N-acylated products there should be two peaks of 1H intensity, one at 2.5–3.2 ppm (alcoholic proton), the other at 6.0–6.5 ppm (amide proton). For O-acylated there should be one peak of 2H intensity (amine protons). Again for the N-acylated product, one peak of intensity 2H at 3.5–3.8 ppm (N-CH_2_ protons) is expected, while for the O-acylated product there should be one peak of intensity 2H at 4.1–4.3 ppm (OC-O-CH_2_ protons). For all our products, we found only N-acylated products were formed. The progress of reaction was monitored throughout the reaction time by HPLC as well as TLC. It was found that only one product was formed after purification, identified as the N-acylated product.

Two alternative routes for synthesis of *N*-acylethanolamine have been investigated by direct acylation with fatty acids **1a**–**d** or transacylation using the simple ester of the fatty acids **1e**–**h** and the optimum conditions for each approach is elucidated. The rates of the reaction and their maximum volumetric productivities depend on the proportions of the substrate ratio.

### Conventional versus microwave irradiation

**Figure 1:**
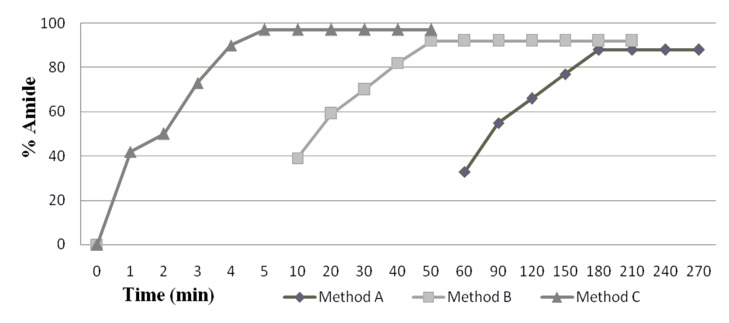
Effect of different methods using 20 mg of enzyme at 60 °C; Method A: conventional method, Method B: solution phase method, Method C: solid support method.

Enzymatic reaction was performed under conventional heating as well as in the presence of microwave irradiation. Considering that immobilized enzymes are highly thermo stable, it is natural that microwave-assistance should also be explored for enzymatic catalysis. It was found that the overall conversion as well as rate of reaction was higher under microwave irradiation than conventional heating. All the parameters were varied under microwave irradiation as well as conventional method. Microwave irradiation gave excellent yield with less reaction time, taking only 4–5 min on solid support and 40–50 min in solution phase while low yields were obtained conventionally even after 4–5 h (Figure 1). In the microwave, the heating is homogenous whereas conventional energy transfer through contact, is comparatively slow and not so effective [23]. Further, a control set with no Novozym^®^ 435 was also irradiated under microwave irradiation, but no reaction occurred (Table 1 and Table 2).

**Table 1:** Comparison of yields and time for the synthesis of hydroxy-substituted aliphatic amide using fatty acids R^1^ = H.

Entry	Compound	Chain Length	Method A^a^ Time (min)/Yield (%)	Method B^b^ Time (min)/Yield (%)	Method C^c^ Time (min)/Yield (%)

1	**3a**	C10	210/78	55/82	5.5/83
2	**3b**	C12	180/87	50/92	5/97
3	**3c**	C14	150/89	45/94	4.5/97.2
4	**3d**	C16	120/92	40/95	4/97.3

^a^conventional method, ^b^solution phase method, ^c^solid support method.

**Table 2:** Comparison of yields and time for the synthesis of hydroxy-substituted aliphatic amide using fatty acid ethyl ester R^1^ = C_2_H_5_.

Entry	Compound	Chain Length	Method A^a^ Time (h)/ Yield (%)	Method B^b^ Time (min)/Yield (%)	Method C^c^ Time (min)/Yield (%)

1	**3a**	C10	210/79	55/83	5.5/84
2	**3b**	C12	180/89	50/94	5/97.2
3	**3c**	C14	150/91	45/95	4.5/97.3
4	**3d**	C16	120/93	40/96.3	4/97.5

^a^conventional method, ^b^solution phase method, ^c^solid support method.

There is remarkable enhancement in reusability of biocatalyst. Conventionally there is a decrease in enzyme activity after run III while in solution phase and solid support there is negligible decrease in activity even in run IV (Table 3).

**Table 3:** Reusability of biocatalyst after I, II, III run.

Run	Method A^a^ Yield	Method B^b^ Yield	Method C^c^ Yield

I (Fresh)	87	92	97
II	78	88	96
III	65	85	95
IV	-	80	93

^a^conventional method, ^b^solution phase method, ^c^solid support method.

### Microwave assisted solid media versus microwave-assisted solution phase

Better results were obtained with the solid support in comparison to solution phase. Under microwave irradiation, the solid support acts as medium to transfer energy, leading to a significant improvement in reactivity. The reason can be attributed to the fact that microwave irradiation coupled with solid support facilitate the evaporation of by products H_2_O/EtOH shifting the equilibrium in the forward direction (Table 4 and Table 5) [24]. In solution phase, solvent used is 1,4-dioxane, which is non polar (log p = −1.1) and make reaction sluggish in nature. The progress of the reaction is due to polarity of reactants and products ethanolamine **2**, fatty acids or esters **1a**–**h** and N-acylated products **3a**–**d**.

### Choice of solvent

The synthesis of *N*-lauroylethanolamine (**3b**) from lauric acid (**1b**) and ethanolamine (**2**) was taken as model reaction to standardize the reaction conditions. Though solvents like diisopropyl ether, acetonitrile, diethyl ether, *tert*-butyl alcohol and 1,4-dioxane are favourable in enzymatic reactions. 1,4-dioxane was found to be the best in terms of solubility of reactants (partially soluble at room temperature and completely soluble at 50 °C) and yield of products.

### Effect of mole ratio of reactants

The effect of mole ratio of fatty acid [lauric acid (**1b**)] and ethanolamine was studied by using 1,4 dioxane with Novozym^®^ 435. In this set of experiments, molar ratios of lauric acid (fatty acid **1b**) to ethanolamine were varied from 1:1/3 to 1:3, where the amount of ethanolamine was kept constant at 2.5 mmol. It was experimentally concluded that the rate of reaction as well as the overall conversion is best in the case of equimolar ratio.

An important aspect governed by mole ratio is pH of the medium and the activity of the enzyme. Higher ratios of ethanolamine to acids led to an excessive shift of the pH away from the optimum value for the lipase, which deactivates the enzyme and hence gives lower yields with higher mole ratio of ethanolamine. Moreover the N-acylation reaction is favoured for mixtures of precursor reagents whose pH values of mixture exceed the p*K*_b_ of ethanolamine (i.e. 4.5). The case of an equimolar ratio of the substrates provide the best condition in terms of two factors - the enzyme activity (the enzyme is deactivated at very high and low pH) and the reactivity of the amino residue [25] (Table 4).

**Table 4:** Effect of the molar ratio using 20 mg of enzyme at 60 °C.

Molar Ratio (Acid/Amine)	Method A^a^ Yield	Method B^b^ Yield	MethodC^c^ Yield

1/0.33	11	12	12
1/0.5	11.5	12	12
1/1	87	92	97
1/2	11.5	12	12
1/3	11	12	12

^a^conventional method, ^b^solution phase method, ^c^solid support method.

### Effect of amount of biocatalyst

Varying the amount of enzyme showed an effect on the rate of the reaction. Initially, 10 mg of enzyme was checked for sufficient catalytic activity. The second set of results, employing 20 mg of enzyme, showed better results than the first. In this case, the rate of conversion of fatty acid and ethanolamine to N-acylated product increased decreasing the reaction time and showing an increase in the initial rate of reaction of fatty acid and ethanolamine. Further loading of more enzyme i.e. 40 mg increased the initial rate of conversion of fatty acid and ethanolamine to N-acylation product. However, after some time the total conversion became constant. Thus 20 mg was found to be the optimal amount and the rest of the experiments were continued with this quantity (Table 5).

**Table 5:** Effect of the amount biocatalyst of using equimolar ratio at 60 °C.

Enzyme (mg)	Method A^a^ Yield	Method B^b^ Yield	Method C^c^ Yield

10	76	83	88
20	87	92	97
30	88	92	98
40	88	92	98

^a^conventional method, ^b^solution phase method, ^c^solid support method.

### Effect of temperature

**Figure 2:**
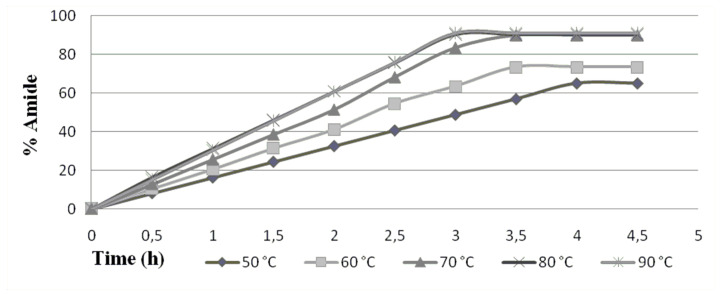
Effect of the temperature using equimolar ratio and 20 mg of enzyme.

The influence of temperature on enzymatic activity was studied by varying the temperature of the reaction from 50–90 °C (method A was employed): further increase in temperature (above 90 °C) leads to deactivation of enzyme (Figure 2).

### Reusability of biocatalyst

Any reusable biocatalyst obviously impacts upon an economic process. A study was performed to determine enzyme activity in terms of reusability. The reusability of enzyme was checked in all methods, for the synthesis of *N*-lauroylethanolamine (**3b**) under optimized conditions as described above. Many experiments were run with the same enzyme, which was filtered, washed with hot 1,4-dioxane and dried in air at room temperature after each run and then reused. There was a marginal decrease in activity in run III or IV, which might be due to handling (Table 3).

### Effect of the chain length of the fatty acid

The reaction rate between ethanolamine (**2**) and fatty acids **1a**–**d** with different chain length (C10–C16) was studied in dioxane at 60 °C using equimolar concentration of both substrates. The experimental results indicate that for long chain fatty acids **1a**–**d**, the rates are faster than for short chain species. The time required for complete conversion of reagent decreases from C10 to C16. The solubilities of the fatty acids **1a**–**d** increase with chain length because a decrease in polarity makes the acid more soluble in the less polar solvent 1,4-dioxane. The highest selectivity was obtained with longest fatty acid (C16) as a consequence of the highest solubility of the corresponding fatty acid (Table 1).

### Effect of transacylation

Transacylation is more selective for formation of the N-acylation product and produces higher conversions than direct acylation. To our surprise, this reaction provides an alternative approach to the direct N-acylation of ethanolamine (**2**) with free fatty acids. Free fatty acids would be more desirable for use as precursor reagents than the corresponding alkyl esters as free fatty acids carry following benefits (i) they are obtained directly by hydrolysis of naturally occurring fats and oils; (ii) they are widely available at low cost and (iii) they do not generate by-products (namely, short chain alcohols) which reduce the activity of the biocatalyst and need to be separated from the desired product (Table 2).

## Conclusion

The combination of microwave-assisted solid support in the enzymatic-catalyzed synthesis of hydroxy-substituted aliphatic amide media was studied. There is significant enhancement in rate of the reaction under microwave irradiation in comparison with conventional heating. It has been experimentally proved that the reusability of the enzyme was found to be greater in the case of microwave rather than conventional heating. This study not only gives wide scope of varying reaction conditions in term of temperature, molar ratio, enzyme ratio, enzyme reusability, fatty acid length, transacylation, phase *etc*. but also provides experimental data for further research work.

## Experimental

### General

^1^H NMR and ^13^C NMR spectra were recorded on Bruker TOP SPIN 300 MHz and 75.6 MHz spectrometer with chemical shift values (δ) in ppm downfield from TMS using CDCl_3_ as solvent. IR spectra were recorded on a model Perkin–Elmer FTIR-1710 spectrometer using KBr disks. Elemental analysis was performed using Heraeus CHN-Rapid Analyzer. EI mass spectra were recorded on TOF MS mass spectrometer. Melting points were taken on a Thomas-Hoover melting point apparatus and are uncorrected. The purity of compounds were checked on silica gel coated aluminum plates (Merck TLC: mass particle size 10–12 μm; particle distribution 5–20 μm; layer thickness 250 μm; plate height 30 μm). Ethanolamine (99.0% pure), lauric acid (>98.5% pure), capric acid (>95.5% pure), myristic (>98.5% pure) and palmitic acid (>98.5%), ethyl laurate (>95.5% pure), ethyl caprate (>95.5% pure), ethyl myristate (>95.5% pure) and ethyl palmitate (>95.5% pure) were purchased from the company Laboratory Reagent and solvents were of HPLC quality and were supplied by SpectroChem.

### Microwave reactor

The studies were carried out in a microwave reactor (Discover, model CEM-SP1245). The reactor was a fully baffled, cylindrical glass vessel (capacity, 120 mL; ID, 4.5 cm) with provision for mechanical stirring. A standard four-blade pitched turbine impeller (diameter, 1.5 cm) was used for agitation. However, the actual reactor volume exposed to the microwave irradiation was 45 mL with 5.5 cm height. There was no bubble formation. Temperature in the reactor was computer controlled. The quantities of reactant and enzyme for reaction procedure were identical to those used for conventional heating.

### Lipase

A lipase from *Candida antarctica* (Lipase B, a non specific lipase) immobilized on macroporous acrylic resin designated “Novozym^®^ 435”, was a gift from Novozymes, Denmark.

### HPLC

The HPLC system consisted of CLC-ODS (M) column, RID-2AS refractive index detector, Shimadzu LC 4A integrator.

## Synthesis of hydroxy-substituted aliphatic amide

### Conventional method

A mixture of fatty acids **1a**–**d** and ethanolamine (**2**) were dissolved in 15 mL of 1,4-dioxane. After becoming clear solution enzyme was added. Then reaction mixture was shaken and heated conventionally for the time indicated in the corresponding experiment. After a time interval of 30 min, the progress of the reaction was checked by TLC in ethanolic system (hexane/ethyl acetate/ethanol = 50:25:25).

### Solution phase method

In an Erlenmeyer flask, fatty acids **1a**–**d** and ethanolamine (**2**) were dissolved in 15 mL of 1,4-dioxane and enzyme was added (same as above). The reaction mixture placed in the microwave and irradiated for 30 s. After time intervals of 2 min, the progress of the reaction was checked in same manner as above.

### Solid support method

In an Erlenmeyer flask, fatty acids **1a**–**d** and ethanolamine (**2**) were dissolved in minimum amount of solvent and impregnated on immobilized enzyme by subsequent evaporation under vacuum. Now enzyme-free carrier material was taken in beaker with dummy load and the reactions were performed in an open vessel. The solid support was treated under microwave irradiation for 10 s. The progress of the reaction was checked at time intervals of 30 s in same manner as above.

### Analysis of reaction mixtures

The course of enzymatic reaction was studied using HPLC. At predetermined reaction times, the fixed amount of reaction mixture (0.5 mL for conventional, 0.1 mL for solution phase and 0.05 g for solid support) was taken out and diluted with methanol and chloroform [50:50 (v/v)]. The immobilized enzyme was removed from reaction mixture via filtration and the reaction mixture made up to the final concentration 2 mM. Calibration analyses were performed using products obtained. The mobile phase, retention times and flow rates used for quantification of different products are given in Table 6.

**Table 6:** Mobile phases, retention time and flow rates for HPLC analyses of the compounds.

Entry	Compound	Mobile Phase (methanol/water)	Time (min)	Flow rate (ml/min)

1	Ethanolamine	90/10	5.4	0.5
2	Amide (C10)	85/15	10.2	0.5
3	Ester (C10)	98/2	5.2	0.5
4	Capric acid	85/15	15.6	0.5
5	Ester (C12)	98/2	5.4	0.5
6	Amide (C12)	90/10	12.8	0.5
7	Lauric acid	98/2	4.4	1.0
8	Amide (C14)	95/5	5.8	1.0
9	Myristic acid	100/0	5.2	1.0
10	Ester (C14)	100/0	3.2	1.0
11	Amide (C16)	98/2	6	1.0
12	Palmitic acid	100/0	6.4	1.0
13	Ester (C16)	100/0	3.4	1.0

### Purification of product

After completion of reaction, the enzyme was removed by filtration and solvent was then removed by evaporation under reduced pressure (for solid support synthesis 15 mL (3 × 5) of 1,4-dioxane was added to dissolved product). The resulting solid was treated with a solution of sodium bicarbonate to remove remaining fatty acid **1a**–**d** and ethanolamine (**2**). The recrystallization was done using methanol and chloroform (50/50 (v/v)).

### *N*-Caprinoylethanolamine (**3a**)

mp 50–54 °C, white crystalline solid, Anal. Calculated for C_12_H_25_NO_2_ C 66.93, H 11.70, N 6.50%, found C 67.28, H 11.83, N 6.54%. IR ν_max_/cm^−1^: 1561 (C-N), 1643 (C=O), 2920 (C-H), 3294 (O-H), ^1^H NMR (300 MHz, CDCl_3_): δ 0.88 (t, *J* = 6.3 Hz, 3H, CH_3_), 1.26 (m, 14H, CH_2_ chain), 2.20 (t, *J* = 7.5 Hz, 2H, CH_2_CO), 3.20 (s, 1H, OH) 3.41 (t, *J* = 4.8 Hz, 2H, CH_2_O), 3.71 (t, *J* = 4.6 Hz, 2H, CH_2_N), 6.20 (s, 1H, NH). ^13^C NMR (75 MHz, CDCl_3_): δ 13.9, 40.2, 41.1, 62.56, 174.3. MS : 214 (M–H)^+^ , 213 (M–H_2_)^+^.

### *N*-Lauroylethanolamine (**3b**)

mp 74–78 °C, white crystalline solid. Anal. Calculated for C_14_H_29_NO_2_ C 69.09, H 12.01, N 5.75, found C 68.38, H 11.93, N 5.54. IR ν_max_/cm^−1^: 1559 (C-N), 1643 (C=O), 2919 (C-H), 3297 (O-H), ^1^H NMR (300 MHz, CDCl_3_): δ 0.87 (t, *J* = 6.3 Hz, 3H, CH_3_), 1.25 (m, 18H, CH_2_ chain), δ 2.20 (t, *J* = 7.8 Hz, 2H, CH_2_CO), 2.67 (s, 1H, OH), 3.27 (t, *J* = 4.8 Hz, 2H, CH_2_O), 3.55 (t, *J* = 4.8 Hz, 2H, CH_2_N), 6.58 (s, 1H, NH). ^13^C NMR (75 MHz, CDCl_3_): δ 13.9, 40.4, 42.1, 61.6, 174.0, MS: 241 (M–H_2_)^+^.

### *N*-Myristoylethanolamine (**3c**)

mp 82–86 °C, white crystalline solid, Anal. Calculated for C_16_H_33_NO_2_ C 70.80, H 12.25, N 5.16, found C 70.68, H 12.35, N 5.14%. IR ν_max_/cm^−1^: 1560 (C-N), 1643 (C=O), 2920 (C-H), 3293 (O-H), ^1^H NMR (300 MHz, CDCl_3_): δ 0.84 (t, *J* = 5.5 Hz, 3H, CH_3_), 1.22 (m, 22H, CH_2_ chain), 2.03 (t, *J* = 7.3 Hz, 2H, CH_2_CO), 2.54 (s, 1H, OH), 3.24 (t, *J* = 5.9 Hz, 2H, CH_2_O), 3.61 (t, *J* = 5.8 Hz, 2H, CH_2_N), 6.42 (s, 1H, NH). ^13^C NMR (75 MHz, CDCl_3_): δ 14.9, 40.8, 41.9, 62.0, 173.4. MS: 271 (M–H)^+^, 213 (M–H_2_)^+^.

### *N*-Palmitoylethanolamine (**3d**)

mp 112–114 °C, white crystalline solid, Anal. Calculated for C_18_H_37_NO_2_ C 72.19, H 12.45, N 4.68%, found C 72.24, H 12.56, N 4.78%. IR ν_max_/cm^−1^: 1558 (C-N), 1643 (C=O), 2850 (C-H), 3298 (O-H), ^1^H NMR (300 MHz, CDCl_3_): δ 0.88 (t, *J* = 6.5 Hz, 3H, CH_3_), 1.23 (m, 24H, CH_2_ chain), 2.12 (t, *J* = 8.2 Hz, 2H, CH_2_CO), 2.62 (s, 1H, OH), 3.42 (t, *J* = 5.2 Hz, 2H, CH_2_O), 3.65 (t, *J* = 5.3 Hz, 2H, CH_2_N), 6.67 (s, 1H, NH). ^13^C NMR (75 MHz, CDCl_3_): δ 13.9, 40.4, 42.1, 61.6, 174.0. MS: 298 (M–H)^+^, 297 (M–H_2_)^+^.
